# Pruritic eruption on the extremities: Report of 2 generations treated with dupilumab

**DOI:** 10.1016/j.jdcr.2025.08.039

**Published:** 2025-09-24

**Authors:** Anna-Marie Hosking, Lina Saeed, Janellen Smith

**Affiliations:** Department of Dermatology, University of California, Irvine, 118 Med Surge I, Irvine, CA, 92697

**Keywords:** basement membrane zone, dupilumab, epidermolysis bullosa, epidermolysis bullosa pruriginosa, pruritus

A 58-year-old Vietnamese male presented with a pruritic eruption on the extremities since the age of 5. Examination revealed diffuse pink lichenified, prurigo-like papules and plaques in a linear distribution on the extremities with associated skin atrophy, scarring, and nail dystrophy (Fig 1). His 10-year-old son also had similar cutaneous findings (Fig 2), and punch biopsies of both patients showed a subepidermal blister and scar, with scant eosinophils (Fig 3); direct immunofluorescence (DIF) was negative. Genetic testing was positive for a heterozygous pathogenic variant in type VII collagen, COL7A1 (c.5264G>A), in both the father and son. They were started on dupilumab with significant clinical improvement and a rapid decrease in pruritus, with a sustained response over 12 months (Fig 4).

**Figure 1:**
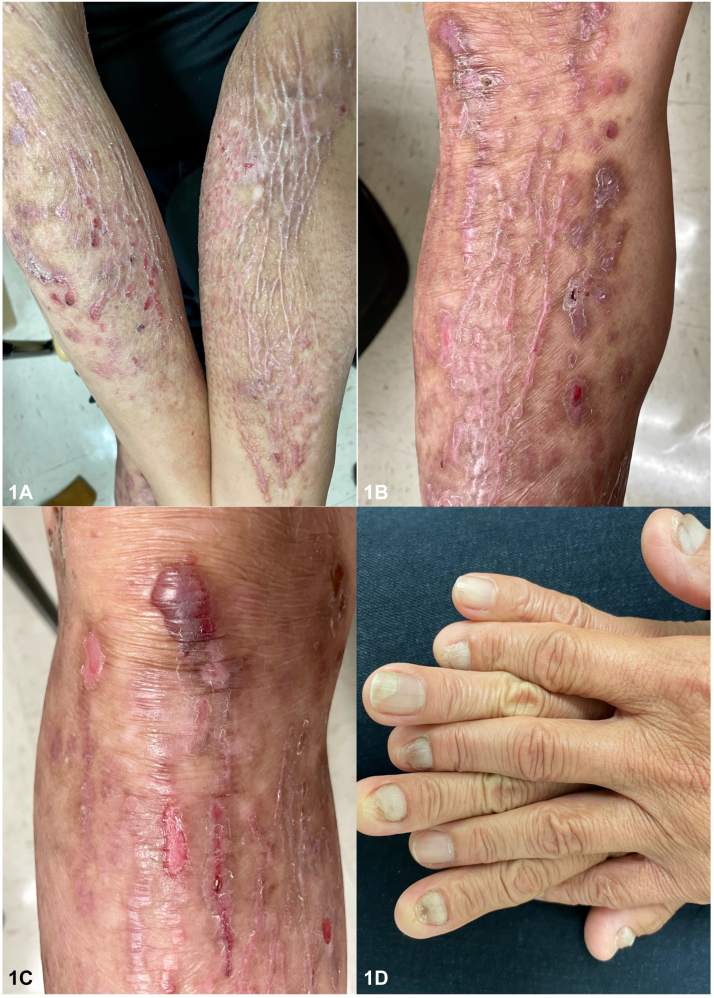

**Figure 2:**
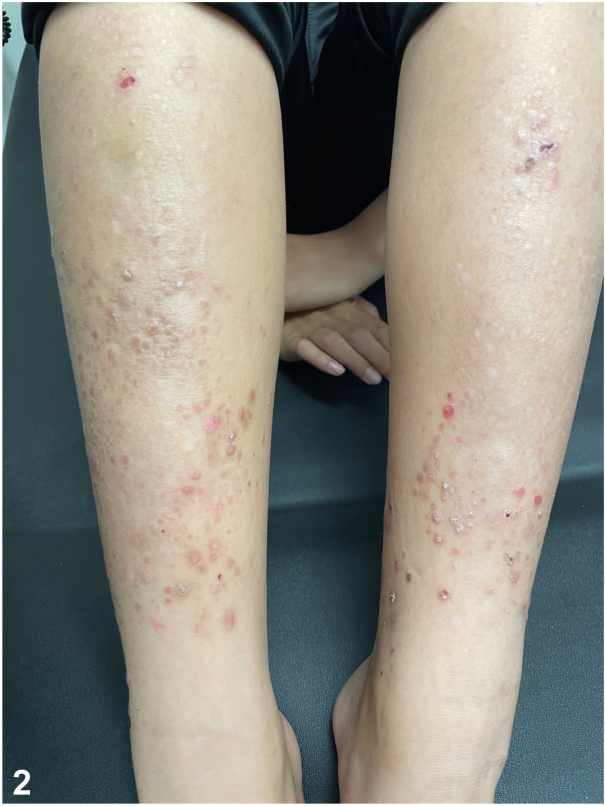

**Figure 3:**
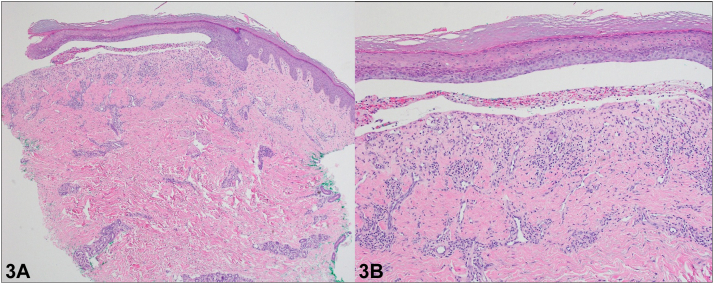

**Figure 4:**
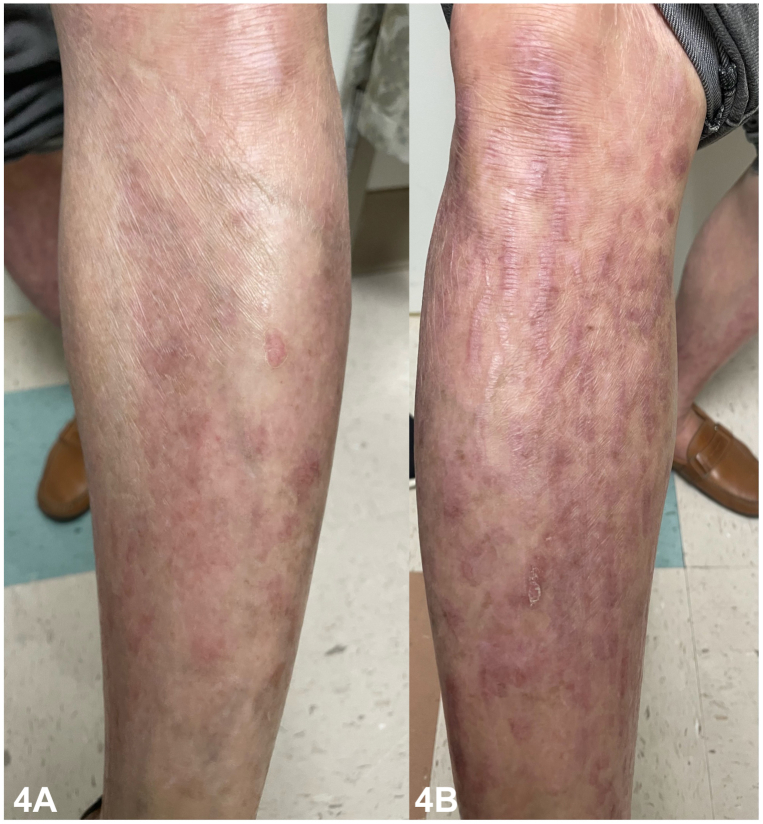

**Question 1: What is your diagnosis?**

A. Bullous pemphigoid
B. Bullous lupus
C. Epidermolysis bullosa simplex
D. Epidermolysis bullosa pruriginosa (EBP)
E. Porphyria cutanea tarda

**Answers:**

**A.** Bullous pemphigoid – Incorrect. Bullous pemphigoid is an autoimmune blistering disorder caused by autoantibodies targeted against bullous pemphigoid antigen 1 and bullous pemphigoid antigen 2. Histopathology would show a subepidermal blister with eosinophils; DIF would be positive for IgG and C3 in a linear pattern at the basement membrane zone (BMZ) in an N-serrated pattern. Immunofluorescence on salt-split skin would localize to the roof.
**B.** Bullous lupus – Incorrect. Bullous lupus is caused by circulating antibodies targeted against collagen VII; however, histopathology would show a subepidermal blister with neutrophils. DIF would show a “full-house pattern” with granular deposition of IgG, IgA, immunoglobulin (Ig) M, and C3 at the BMZ in a U-serrated pattern. Collagen VII is the target epitope in epidermolysis bullosa acquisita, a rare, acquired subepidermal bullous disease. Classically, epidermolysis bullosa acquisita shows a cell-poor subepidermal blister on histopathology. DIF would show a broad linear band of IgG>C3 in a U-serrated pattern along the BMZ. Immunofluorescence on salt-split skin in both disorders would localize to the floor.
**C.** Epidermolysis bullosa simplex – Incorrect. Epidermolysis bullosa simplex is an inherited blistering disorder caused by mutations in genes encoding keratin 5 and keratin 14, resulting in tissue fragility.^1^ Histopathology would show an intraepidermal split. DIF would be negative.
**D.** EBP – Correct. EBP is a rare subtype of inherited dystrophic epidermolysis bullosa (DEB) characterized by extremely pruritic lichenified, prurigo-like plaques and nodules. Pretibial involvement is typical, but it can also involve the forearms and trunk; nail dystrophy is common.^2^ Histopathology shows hyperkeratosis, acanthosis, and subepidermal cleavage. DIF is negative.^3^, ^4^, ^5^
**E.** Porphyria cutanea tarda – Incorrect. Porphyria cutanea tarda is a blistering disorder caused by a deficiency of uroporphyrinogen III decarboxylase. Histopathology would show a subepidermal noninflammatory blister with festooning, caterpillar bodies, and periodic acid–Schiff + perivascular hyaline material. DIF would show IgM + C3 around vessels.

**Question 2: Collagen VII comprises which of the following components of the basement membrane?**

A. Intermediate filaments
B. Desmosomes
C. Anchoring fibrils
D. Focal adhesions
E. Hemidesmosomes

**Answers:**

**A.** Intermediate filaments – Incorrect. Intermediate filaments are comprised of keratins 5 and 14 in the basal keratinocyte. They provide tensile strength via a mesh–like cytoskeletal network and attach to hemidesmosomes on the basal surface of the keratinocyte.
**B.** Desmosomes – Incorrect. Desmosomes are intercellular junctions comprised of cadherins (desmogleins and desmocollins), armadillo proteins (plakogloblin and plakophilins), and desmoplakin and provide cell–cell adhesion.
**C.** Anchoring fibrils – Correct. Anchoring fibrils are composed of type VII collagen dimers that interact with types I, III, and IV collagen as well as laminins and integrins and form the inferior component of the cutaneous BMZ adhesion complex. Mutations in collagen VII are responsible for the dominant dystrophic subtype of epidermolysis bullosa, including EBP.
**D.** Focal adhesions – Incorrect. Focal adhesions consist of adhesion proteins (kindlin, actin, talin, and vinculin) that contribute to integrin activation through binding of integrin-β tails.
**E.** Hemidesmosomes – Incorrect. Hemidesmosomes are comprised of an inner and outer plaque consisting of multiple elements, including bullous pemphigoid antigen 1, bullous pemphigoid antigen 2, plectin, and integrin α6β4.

**Question 3: What is the mechanism of action of the medication that was recently Food and Drug Administration-approved for the treatment of DEB?**

A. Inhibits interleukin IL-12 and IL-23
B. Inhibits IgE
C. Inhibits IL-13 and IL-4
D. Inhibits phosphodiesterase 4
E. Modified herpes simplex virus type 1 (HSV-1) vector

**Answers:**

**A.** Inhibits 1L-12 and IL-23 – Incorrect. Ustekinumab targets the P40 subunit of IL-12 and IL-23 and is used in the treatment of psoriasis.
**B.** Inhibits IgE – Incorrect. Omalizumab inhibits IgE and is used in the treatment of asthma and chronic urticaria.
**C.** Inhibits IL-13 and IL-4 – Incorrect. Dupilumab inhibits the alpha subunit of the IL-4 receptor (IL-4Rα), providing dual blockade of both IL-4 and IL-13. Dupilumab is hypothesized to decrease itch in EBP by blocking IL-4Rα-induced sensitization of sensory neurons to pruritogens.
**D.** Inhibits phosphodiesterase 4 – Incorrect. Apremilast is a phosphodiesterase 4 inhibitor approved for the treatment of psoriatic arthritis, plaque psoriasis, and Behçet disease.
**E.** Modified HSV-1 vector – Correct. Beremagene geperpavec is a topical HSV-1–based gene therapy designed to restore C7 protein by delivering *COL7A1.* Beremagene geperpavec was recently Food and Drug Administration-approved in 2023 for the treatment of DEB in patients 6 months or older.

## Conflicts of interest

None disclosed.
